# Supplementary material for: Sex- and Mutation-Specific p53 Gain-of-Function Activity in Gliomagenesis
Source: Cancer Res Commun. 2021 Dec 15;1(3):148–63. doi: 10.1158/2767-9764.CRC-21-0026 (PMC8694557; doi:10.1158/2767-9764.CRC-21-0026)
Supplement: Supplementary Figures 1-5, Tables 1-7 [file crc-21-0026-s01.docx]

**Supplemental Table 1.** Aggregated studies for p53 mutation analysis.

| **Cancer group** | **Studies** | **Cancer Types** | **Total number of tumors** |
| --- | --- | --- | --- |
| **Pan Cancer** | MSK-IMPACT Clinical Sequencing Cohort (MSKCC, Nat Med 2017)^1^ | Adenocarcinoma, Adenocarcinoma of Lung, Adenocarcinoma, Mucinous, Breast Neoplasms, Carcinoma, Ductal, Breast, Carcinoma, Hepatocellular, Carcinoma, Lobular, Carcinoma, Non-Small-Cell Lung, Carcinoma, Renal Cell, Carcinoma, Squamous Cell, Carcinosarcoma, Cholangiocarcinoma, Colonic Neoplasms, Colorectal Neoplasms, Cystadenocarcinoma, Serous, DNA Mutational Analysis, Gallbladder Neoplasms, Glioblastoma, Liposarcoma, Lung Neoplasms, Lymphoma, Follicular, Melanoma, Mesothelioma, Neoplasms, Squamous Cell, Neurofibroma, Oligodendroglioma, Osteosarcoma, Ovarian Neoplasms, Pancreatic Neoplasms, Prostatic Neoplasms, Rectal Neoplasms, Sarcoma, Sarcoma, Synovial, Squamous Cell Carcinoma of Head and Neck, Stomach Neoplasms, Thyroid Cancer, Papillary, Urinary Bladder Neoplasms, Uterine Neoplasms, Uveal Neoplasms | 4985 |
| **Breast Cancer** | Breast Cancer (METABRIC, Nature 2012 & Nat Commun 2016)^2,3^, Breast Cancer (MSK, Cancer Cell 2018)^4^, Breast Cancer (MSK, Nature Cancer 2020)^5^, Breast Cancer (MSKCC, NPJ Breast Cancer 2019)^6^, Breast Cancer (SMC 2018)^7^, Breast Cancer Xenografts (British Columbia, Nature 2015)^8^, Breast Fibroepithelial Tumors (Duke-NUS, Nat Genet 2015)^9^, Breast Invasive Carcinoma (British Columbia, Nature 2012)^10^, Breast Invasive Carcinoma (Broad, Nature 2012)^11^, Breast Invasive Carcinoma (TCGA, Cell 2015)^12^, Breast Invasive Carcinoma (TCGA, Firehose Legacy), Breast Invasive Carcinoma (TCGA, Nature 2012)^13^, Breast Invasive Carcinoma (TCGA, PanCancer Atlas)^14^, Metastatic Breast Cancer (INSERM, PLoS Med 2016)^15^, The Metastatic Breast Cancer Project (Provisional, February 2020) | Breast, Breast Invasive Cancer, NOS, Breast Invasive Carcinoma, Breast Invasive Carcinoma (NOS), Breast Invasive Ductal Carcinoma, Breast Invasive Mixed Mucinous Carcinoma, Breast Mixed Ductal and Lobular Carcinoma, Ductal Carcinoma In Situ (DCIS), Infiltrating Ductal Carcinoma, Infiltrating Lobular Carcinoma, Invasive Breast Cancer, Invasive Breast Carcinoma, Malignant Phyllodes Tumor of the Breast, Medullary Breast Carcinoma, Metaplastic Breast Cancer, Mixed Carcinoma, Paget Disease of the Nipple | 3219 |
| **Lung Cancer** | Lung Adenocarcinoma (Broad, Cell 2012)^16^, Lung Adenocarcinoma (MSKCC, Science 2015)^17^, Lung Adenocarcinoma (OncoSG, Nat Genet 2020)^18^, Lung Adenocarcinoma (TCGA, Firehose Legacy), Lung Adenocarcinoma (TCGA, Nature 2014)^19^, Lung Adenocarcinoma (TCGA, PanCancer Atlas), Lung Adenocarcinoma (TSP, Nature 2008)^20^, Lung Cancer (SMC, Cancer Research 2016)^21^, Lung Squamous Cell Carcinoma (TCGA, Firehose Legacy), Lung Squamous Cell Carcinoma (TCGA, Nature 2012), Lung Squamous Cell Carcinoma (TCGA, PanCancer Atlas), Non-Small Cell Cancer (MSKCC, Cancer Discov 2017)^22^, Non-small cell lung cancer (MSK, Science 2015)^17^, Non-Small Cell Lung Cancer (MSKCC, J Clin Oncol 2018)^23^, Non-Small Cell Lung Cancer (TRACERx, NEJM & Nature 2017)^24^, Non-Small Cell Lung Cancer (University of Turin, Lung Cancer 2017)^25^, Pan-Lung Cancer (TCGA, Nat Genet 2016)^26^, Small Cell Lung Cancer (CLCGP, Nat Genet 2012)^27^, Small Cell Lung Cancer (Johns Hopkins, Nat Genet 2012)^28^, Small Cell Lung Cancer (U Cologne, Nature 2015)^29^, Small-Cell Lung Cancer (Multi-Institute, Cancer Cell 2017)^30^, Thoracic PDX (MSK, Provisional) | Combined Small Cell Lung Carcinoma, Large Cell Lung Carcinoma, Large Cell Neuroendocrine Carcinoma, Lung Adenocarcinoma, Lung Adenosquamous Carcinoma, Lung Neuroendocrine Tumor, Lung Squamous Cell Carcinoma, Non-Small Cell Lung Cancer, Pleomorphic Carcinoma of the Lung, Pleural Mesothelioma, Poorly Differentiated Non-Small Cell Lung Cancer, Sarcomatoid Carcinoma of the Lung, Small Cell Lung Cancer | 3727 |
| **Skin** | Acral Melanoma (TGEN, Genome Res 2017)^31^, Desmoplastic Melanoma (Broad Institute, Nat Genet 2015)^32^, Melanoma (Broad/Dana Farber, Nature 2012)^33^, Melanoma (MSKCC, NEJM 2014)^34^, Melanomas (TCGA, Cell 2015)^35^, Metastatic Melanoma (DFCI, Nature Medicine 2019)^36^, Metastatic Melanoma (DFCI, Science 2015)^37^, Metastatic Melanoma (MSKCC, JCO Precis Oncol 2017)^38^, Metastatic Melanoma (UCLA, Cell 2016)^39^, Skin Cutaneous Melanoma (Broad, Cell 2012)^40^, Skin Cutaneous Melanoma (TCGA, Firehose Legacy), Skin Cutaneous Melanoma (TCGA, PanCancer Atlas)^14^, Skin Cutaneous Melanoma (Yale, Nat Genet 2012)^41^, Skin Cutaneous Melanoma(Broad, Cancer Discov 2014)^42^ | Acral Melanoma, Cutaneous Melanoma, Desmoplastic Melanoma, Melanoma, Melanoma of Unknown Primary | 257 |
| **Prostate** | Prostate Adenocarcinoma (Broad/Cornell, Cell 2013)^43^, Prostate Adenocarcinoma (Broad/Cornell, Nat Genet 2012)^44^, Prostate Adenocarcinoma (CPC-GENE, Nature 2017)^45^, Prostate Adenocarcinoma (Fred Hutchinson CRC, Nat Med 2016)^46^, Prostate Adenocarcinoma (MSK, Eur Urol 2020)^47^, Prostate Adenocarcinoma (MSKCC, Cancer Cell 2010)^48^, Prostate Adenocarcinoma (MSKCC/DFCI, Nature Genetics 2018)^49^, Prostate Adenocarcinoma (SMMU, Eur Urol 2017)^50^, Prostate Adenocarcinoma (TCGA, Firehose Legacy), Prostate Adenocarcinoma Organoids (MSKCC, Cell 2014)^51^, Prostate Cancer (MSKCC, JCO Precis Oncol 2017)^52^ | Prostate Adenocarcinoma, Prostate Neuroendocrine Carcinoma, Prostate Small Cell Carcinoma | 970 |
| **Colon** | Colon Adenocarcinoma (CaseCCC, PNAS 2015)^53^, Colon Cancer (CPTAC-2 Prospective, Cell 2019)^54^, Colorectal Adenocarcinoma (DFCI, Cell Reports 2016)^55^, Colorectal Adenocarcinoma (Genentech, Nature 2012)^56^, Colorectal Adenocarcinoma (TCGA, Firehose Legacy), Colorectal Adenocarcinoma (TCGA, PanCancer Atlas)^14^, Colorectal Adenocarcinoma Triplets (MSKCC, Genome Biol 2014)^57^, Metastatic Colorectal Cancer (MSKCC, Cancer Cell 2018)^58^, Rectal Cancer (MSK,Nature Medicine 2019)^59^ | Colorectal Adenocarcinoma, Colon Adenocarcinoma, Rectal Adenocarcinoma, Mucinous Adenocarcinoma | 2342 |
| **CNS** | Anaplastic Oligodendroglioma and Anaplastic Oligoastrocytoma (MSKCC, Neuro Oncol 2017)^60^, Brain Lower Grade Glioma (TCGA, Firehose Legacy), Brain Tumor PDXs (Mayo Clinic, 2019), Glioblastoma (Columbia, Nat Med. 2019)^61^, Glioblastoma (TCGA, Cell 2013)^62^, Glioblastoma (TCGA, Nature 2008)^63^, Glioblastoma Multiforme (TCGA, Firehose Legacy), Glioma (MSK, Nature 2019)^64^, Glioma (MSKCC, Clin Cancer Res 2019)^65^, Low-Grade Gliomas (UCSF, Science 2014)^66^, Merged Cohort of LGG and GBM (TCGA, Cell 2016)^67^, Pheochromocytoma and Paraganglioma (TCGA, Firehose Legacy) | Anaplastic Astrocytoma, Anaplastic Oligoastrocytoma, Anaplastic Oligodendroglioma, Astrocytoma, Desmoplastic/Nodular Medulloblastoma, Diffuse Astrocytoma, Diffuse Glioma, Glioblastoma, Glioblastoma Multiforme, Gliosarcoma, High-Grade Glioma, NOS, Large Cell/Anaplastic Medulloblastoma, Low-Grade Glioma (NOS), Medulloblastoma, Oligoastrocytoma, Oligodendroglioma, Pheochromocytoma, Rosette-forming Glioneuronal Tumor of the Fourth Ventricle | 1979 |
| **Glioblastoma** | Glioblastoma (Columbia, Nat Med. 2019)^68^, Glioblastoma (TCGA, Cell 2013)^62^, Glioblastoma Multiforme (TCGA, Firehose Legacy) | Glioblastoma Multiforme | 316 |

**Supplementary Table 1 References**

1. Zehir A, Benayed R, Shah RH, et al. Mutational landscape of metastatic cancer revealed from prospective clinical sequencing of 10,000 patients. *Nat Med*. 2017;23(6):703-713. doi:10.1038/nm.4333

2. Pereira B, Chin SF, Rueda OM, et al. The somatic mutation profiles of 2,433 breast cancers refines their genomic and transcriptomic landscapes. *Nat Commun*. 2016;7. doi:10.1038/ncomms11479

3. Curtis C, Shah SP, Chin SF, et al. The genomic and transcriptomic architecture of 2,000 breast tumours reveals novel subgroups. *Nature*. 2012;486(7403):346-352. doi:10.1038/nature10983

4. Razavi P, Chang MT, Xu G, et al. The Genomic Landscape of Endocrine-Resistant Advanced Breast Cancers. *Cancer Cell*. 2018;34(3):427-438.e6. doi:10.1016/j.ccell.2018.08.008

5. Razavi P, Dickler MN, Shah PD, et al. Alterations in PTEN and ESR1 promote clinical resistance to alpelisib plus aromatase inhibitors. *Nat Cancer*. 2020;1(4):382-393. doi:10.1038/s43018-020-0047-1

6. Nixon MJ, Formisano L, Mayer IA, et al. PIK3CA and MAP3K1 alterations imply luminal A status and are associated with clinical benefit from pan-PI3K inhibitor buparlisib and letrozole in ER+ metastatic breast cancer. *npj Breast Cancer*. 2019;5(1). doi:10.1038/s41523-019-0126-6

7. Kan Z, Ding Y, Kim J, et al. Multi-omics profiling of younger Asian breast cancers reveals distinctive molecular signatures. *Nat Commun*. 2018;9(1). doi:10.1038/s41467-018-04129-4

8. Eirew P, Steif A, Khattra J, et al. Dynamics of genomic clones in breast cancer patient xenografts at single-cell resolution. *Nature*. 2015;518(7539):422-426. doi:10.1038/nature13952

9. Tan J, Ong CK, Lim WK, et al. Genomic landscapes of breast fibroepithelial tumors. *Nat Genet*. 2015;47(11):1341-1345. doi:10.1038/ng.3409

10. Shah SP, Roth A, Goya R, et al. The clonal and mutational evolution spectrum of primary triple-negative breast cancers. *Nature*. 2012;486(7403):395-399. doi:10.1038/nature10933

11. Banerji S, Cibulskis K, Rangel-Escareno C, et al. Sequence analysis of mutations and translocations across breast cancer subtypes. *Nature*. 2012;486(7403):405-409. doi:10.1038/nature11154

12. Ciriello G, Gatza ML, Beck AH, et al. Comprehensive Molecular Portraits of Invasive Lobular Breast Cancer. *Cell*. 2015;163(2):506-519. doi:10.1016/j.cell.2015.09.033

13. Koboldt DC, Fulton RS, McLellan MD, et al. Comprehensive molecular portraits of human breast tumours. *Nature*. 2012;490(7418):61-70. doi:10.1038/nature11412

14. Hoadley KA, Yau C, Hinoue T, et al. Cell-of-Origin Patterns Dominate the Molecular Classification of 10,000 Tumors from 33 Types of Cancer. *Cell*. 2018;173(2):291-304.e6. doi:10.1016/j.cell.2018.03.022

15. Lefebvre C, Bachelot T, Filleron T, et al. Mutational Profile of Metastatic Breast Cancers: A Retrospective Analysis. *PLoS Med*. 2016;13(12). doi:10.1371/journal.pmed.1002201

16. Imielinski M, Berger AH, Hammerman PS, et al. Mapping the hallmarks of lung adenocarcinoma with massively parallel sequencing. *Cell*. 2012;150(6):1107-1120. doi:10.1016/j.cell.2012.08.029

17. Rizvi NA, Hellmann MD, Snyder A, et al. Mutational landscape determines sensitivity to PD-1 blockade in non-small cell lung cancer. *Science (80- )*. 2015;348(6230):124-128. doi:10.1126/science.aaa1348

18. Chen J, Yang H, Teo ASM, et al. Genomic landscape of lung adenocarcinoma in East Asians. *Nat Genet*. 2020;52(2):177-186. doi:10.1038/s41588-019-0569-6

19. Collisson EA, Campbell JD, Brooks AN, et al. Comprehensive molecular profiling of lung adenocarcinoma: The cancer genome atlas research network. *Nature*. 2014;511(7511):543-550. doi:10.1038/nature13385

20. Ding L, Getz G, Wheeler DA, et al. Somatic mutations affect key pathways in lung adenocarcinoma. *Nature*. 2008;455(7216):1069-1075. doi:10.1038/nature07423

21. Um SW, Joung JG, Lee H, et al. Molecular evolution patterns in metastatic lymph nodes reflect the differential treatment response of advanced primary lung cancer. *Cancer Res*. 2016;76(22):6568-6576. doi:10.1158/0008-5472.CAN-16-0873

22. Jordan EJ, Kim HR, Arcila ME, et al. Prospective comprehensive molecular characterization of lung adenocarcinomas for efficient patient matching to approved and emerging therapies. *Cancer Discov*. 2017;7(6):596-609. doi:10.1158/2159-8290.CD-16-1337

23. Rizvi H, Sanchez-Vega F, La K, et al. Molecular determinants of response to anti-programmed cell death (PD)-1 and anti-programmed death-ligand 1 (PD-L1) blockade in patients with non-small-cell lung cancer profiled with targeted next-generation sequencing. *J Clin Oncol*. 2018;36(7):633-641. doi:10.1200/JCO.2017.75.3384

24. Jamal-Hanjani M, Wilson GA, McGranahan N, et al. Tracking the Evolution of Non–Small-Cell Lung Cancer. *N Engl J Med*. 2017;376(22):2109-2121. doi:10.1056/nejmoa1616288

25. Vavalà T, Monica V, Lo Iacono M, et al. Precision medicine in age-specific non-small-cell-lung-cancer patients: Integrating biomolecular results into clinical practice—A new approach to improve personalized translational research. *Lung Cancer*. 2017;107:84-90. doi:10.1016/j.lungcan.2016.05.021

26. Campbell JD, Alexandrov A, Kim J, et al. Distinct patterns of somatic genome alterations in lung adenocarcinomas and squamous cell carcinomas. *Nat Genet*. 2016;48(6):607-616. doi:10.1038/ng.3564

27. Peifer M, Fernández-Cuesta L, Sos ML, et al. Integrative genome analyses identify key somatic driver mutations of small-cell lung cancer. *Nat Genet*. 2012;44(10):1104-1110. doi:10.1038/ng.2396

28. Rudin CM, Durinck S, Stawiski EW, et al. Comprehensive genomic analysis identifies SOX2 as a frequently amplified gene in small-cell lung cancer. *Nat Genet*. 2012;44(10):1111-1116. doi:10.1038/ng.2405

29. George J, Lim JS, Jang SJ, et al. Comprehensive genomic profiles of small cell lung cancer. *Nature*. 2015;524(7563):47-53. doi:10.1038/nature14664

30. Gardner EE, Lok BH, Schneeberger VE, et al. Chemosensitive Relapse in Small Cell Lung Cancer Proceeds through an EZH2-SLFN11 Axis. *Cancer Cell*. 2017;31(2):286-299. doi:10.1016/j.ccell.2017.01.006

31. Liang WS, Hendricks W, Kiefer J, et al. Integrated genomic analyses reveal frequent TERT aberrations in acral melanoma. *Genome Res*. 2017;27(4):524-532. doi:10.1101/gr.213348.116

32. Shain AH, Garrido M, Botton T, et al. Exome sequencing of desmoplastic melanoma identifies recurrent NFKBIE promoter mutations and diverse activating mutations in the MAPK pathway. *Nat Genet*. 2015;47(10):1194-1199. doi:10.1038/ng.3382

33. Berger MF, Hodis E, Heffernan TP, et al. Melanoma genome sequencing reveals frequent PREX2 mutations. *Nature*. 2012;485(7399):502-506. doi:10.1038/nature11071

34. Snyder A, Makarov V, Merghoub T, et al. Genetic Basis for Clinical Response to CTLA-4 Blockade in Melanoma. *N Engl J Med*. 2014;371(23):2189-2199. doi:10.1056/nejmoa1406498

35. Akbani R, Akdemir KC, Aksoy BA, et al. Genomic Classification of Cutaneous Melanoma. *Cell*. 2015;161(7):1681-1696. doi:10.1016/j.cell.2015.05.044

36. Liu D, Schilling B, Liu D, et al. Integrative molecular and clinical modeling of clinical outcomes to PD1 blockade in patients with metastatic melanoma. *Nat Med*. 2019;25(12):1916-1927. doi:10.1038/s41591-019-0654-5

37. Van Allen EM, Miao D, Schilling B, et al. Genomic correlates of response to CTLA-4 blockade in metastatic melanoma. *Science (80- )*. 2015;350(6257):207-211. doi:10.1126/science.aad0095

38. Catalanotti F, Cheng DT, Shoushtari AN, et al. PTEN Loss-of-Function Alterations Are Associated With Intrinsic Resistance to BRAF Inhibitors in Metastatic Melanoma . *JCO Precis Oncol*. 2017;1(1):1-15. doi:10.1200/po.16.00054

39. Hugo W, Zaretsky JM, Sun L, et al. Genomic and Transcriptomic Features of Response to Anti-PD-1 Therapy in Metastatic Melanoma. *Cell*. 2016;165(1):35-44. doi:10.1016/j.cell.2016.02.065

40. Hodis E, Watson IR, Kryukov G V., et al. A landscape of driver mutations in melanoma. *Cell*. 2012;150(2):251-263. doi:10.1016/j.cell.2012.06.024

41. Krauthammer M, Kong Y, Ha BH, et al. Exome sequencing identifies recurrent somatic RAC1 mutations in melanoma. *Nat Genet*. 2012;44(9):1006-1014. doi:10.1038/ng.2359

42. Van Allen EM, Wagle N, Sucker A, et al. The genetic landscape of clinical resistance to RAF inhibition in metastatic melanoma. *Cancer Discov*. 2014;4(1):94-109. doi:10.1158/2159-8290.cd-13-0617

43. Baca SC, Prandi D, Lawrence MS, et al. Punctuated evolution of prostate cancer genomes. *Cell*. 2013;153(3):666-677. doi:10.1016/j.cell.2013.03.021

44. Barbieri CE, Baca SC, Lawrence MS, et al. Exome sequencing identifies recurrent SPOP, FOXA1 and MED12 mutations in prostate cancer. *Nat Genet*. 2012;44(6):685-689. doi:10.1038/ng.2279

45. Fraser M, Sabelnykova VY, Yamaguchi TN, et al. Genomic hallmarks of localized, non-indolent prostate cancer. *Nature*. 2017;541(7637):359-364. doi:10.1038/nature20788

46. Kumar A, Coleman I, Morrissey C, et al. Substantial interindividual and limited intraindividual genomic diversity among tumors from men with metastatic prostate cancer. *Nat Med*. 2016;22(4):369-378. doi:10.1038/nm.4053

47. Nguyen B, Mota JM, Nandakumar S, et al. Pan-cancer Analysis of CDK12 Alterations Identifies a Subset of Prostate Cancers with Distinct Genomic and Clinical Characteristics. *Eur Urol*. 2020;78(5):671-679. doi:10.1016/j.eururo.2020.03.024

48. Taylor BS, Schultz N, Hieronymus H, et al. Integrative Genomic Profiling of Human Prostate Cancer. *Cancer Cell*. 2010;18(1):11-22. doi:10.1016/j.ccr.2010.05.026

49. Armenia J, Wankowicz SAM, Liu D, et al. The long tail of oncogenic drivers in prostate cancer. *Nat Genet*. 2018;50(5):645-651. doi:10.1038/s41588-018-0078-z

50. Ren S, Wei GH, Liu D, et al. Whole-genome and Transcriptome Sequencing of Prostate Cancer Identify New Genetic Alterations Driving Disease Progression [Figure presented]. *Eur Urol*. 2018;73(3):322-339. doi:10.1016/j.eururo.2017.08.027

51. Gao D, Vela I, Sboner A, et al. Organoid cultures derived from patients with advanced prostate cancer. *Cell*. 2014;159(1):176-187. doi:10.1016/j.cell.2014.08.016

52. Abida W, Armenia J, Gopalan A, et al. Prospective Genomic Profiling of Prostate Cancer Across Disease States Reveals Germline and Somatic Alterations That May Affect Clinical Decision Making. *JCO Precis Oncol*. 2017;2017(1):1-16. doi:10.1200/po.17.00029

53. Guda K, Veigl ML, Varadan V, et al. Novel recurrently mutated genes in African American colon cancers. *Proc Natl Acad Sci U S A*. 2015;112(4):1149-1154. doi:10.1073/pnas.1417064112

54. Vasaikar S, Huang C, Wang X, et al. Proteogenomic Analysis of Human Colon Cancer Reveals New Therapeutic Opportunities. *Cell*. 2019;177(4):1035-1049.e19. doi:10.1016/j.cell.2019.03.030

55. Giannakis M, Mu XJ, Shukla SA, et al. Genomic Correlates of Immune-Cell Infiltrates in Colorectal Carcinoma. *Cell Rep*. 2016;15(4):857-865. doi:10.1016/j.celrep.2016.03.075

56. Seshagiri S, Stawiski EW, Durinck S, et al. Recurrent R-spondin fusions in colon cancer. *Nature*. 2012;488(7413):660-664. doi:10.1038/nature11282

57. Brannon AR, Vakiani E, Sylvester BE, et al. Comparative sequencing analysis reveals high genomic concordance between matched primary and metastatic colorectal cancer lesions. *Genome Biol*. 2014;15(8). doi:10.1186/s13059-014-0454-7

58. Yaeger R, Chatila WK, Lipsyc MD, et al. Clinical Sequencing Defines the Genomic Landscape of Metastatic Colorectal Cancer. *Cancer Cell*. 2018;33(1):125-136.e3. doi:10.1016/j.ccell.2017.12.004

59. Razavi P, Li BT, Brown DN, et al. High-intensity sequencing reveals the sources of plasma circulating cell-free DNA variants. *Nat Med*. 2019;25(12):1928-1937. doi:10.1038/s41591-019-0652-7

60. Thomas AA, Abrey LE, Terziev R, et al. Multicenter phase II study of temozolomide and myeloablative chemotherapy with autologous stem cell transplant for newly diagnosed anaplastic oligodendroglioma. *Neuro Oncol*. 2017;19(10):1380-1390. doi:10.1093/neuonc/nox086

61. Zhang C, Liu J, Xu D, Zhang T, Hu W, Feng Z. Gain-of-function mutant p53 in cancer progression and therapy. *J Mol Cell Biol*. 2020;12(9):674-687. doi:10.1093/jmcb/mjaa040

62. Brennan CW, Verhaak RGW, McKenna A, et al. The somatic genomic landscape of glioblastoma. *Cell*. 2013;155(2):462. doi:10.1016/j.cell.2013.09.034

63. McLendon R, Friedman A, Bigner D, et al. Comprehensive genomic characterization defines human glioblastoma genes and core pathways. *Nature*. 2008;455(7216):1061-1068. doi:10.1038/nature07385

64. Miller AM, Shah RH, Pentsova EI, et al. Tracking tumour evolution in glioma through liquid biopsies of cerebrospinal fluid. *Nature*. 2019;565(7741):654-658. doi:10.1038/s41586-019-0882-3

65. Jonsson P, Lin AL, Young RJ, et al. Genomic correlates of disease progression and treatment response in prospectively characterized gliomas. *Clin Cancer Res*. 2019;25(18):5537-5547. doi:10.1158/1078-0432.CCR-19-0032

66. Johnson BE, Mazor T, Hong C, et al. Mutational analysis reveals the origin and therapy-driven evolution of recurrent glioma. *Science (80- )*. 2014;343(6167):189-193. doi:10.1126/science.1239947

67. Ceccarelli M, Barthel FP, Malta TM, et al. Molecular Profiling Reveals Biologically Discrete Subsets and Pathways of Progression in Diffuse Glioma. *Cell*. 2016;164(3):550-563. doi:10.1016/j.cell.2015.12.028

68. Zhao J, Chen AX, Gartrell RD, et al. Immune and genomic correlates of response to anti-PD-1 immunotherapy in glioblastoma. *Nat Med*. 2019;25(3):462-469. doi:10.1038/s41591-019-0349-y

**
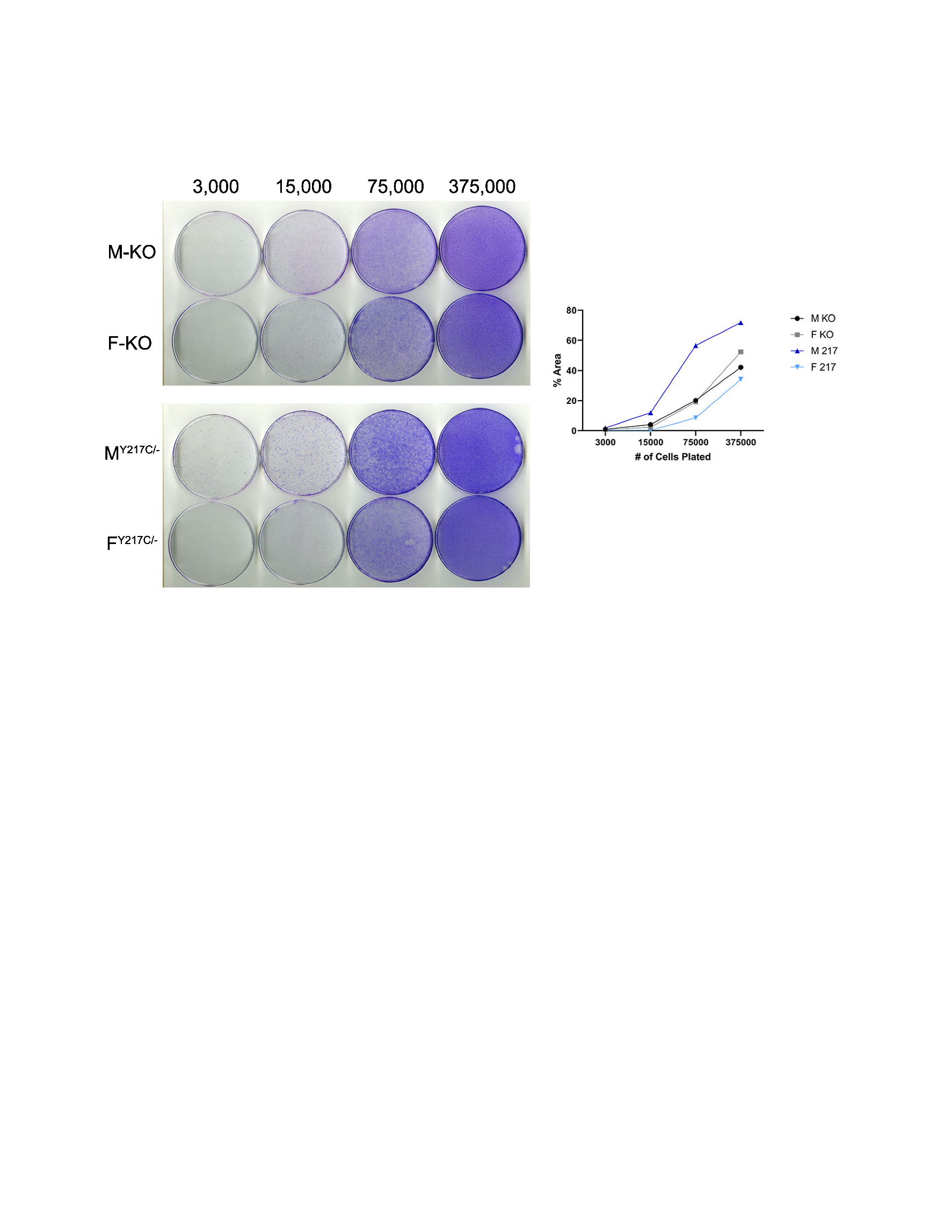
**

**Supplemental Figure 1.** Titration of p53^Y217C^ foci assay. **A.** Male and female p53 KO and p53^Y217C^ astrocytes were plated at a 5-fold dilution in 10 cm plates and incubated for five days before fixing and staining and with Giemsa stain. **B.** Quantification of foci assay as measured by % area covered by stained nuclei.

**
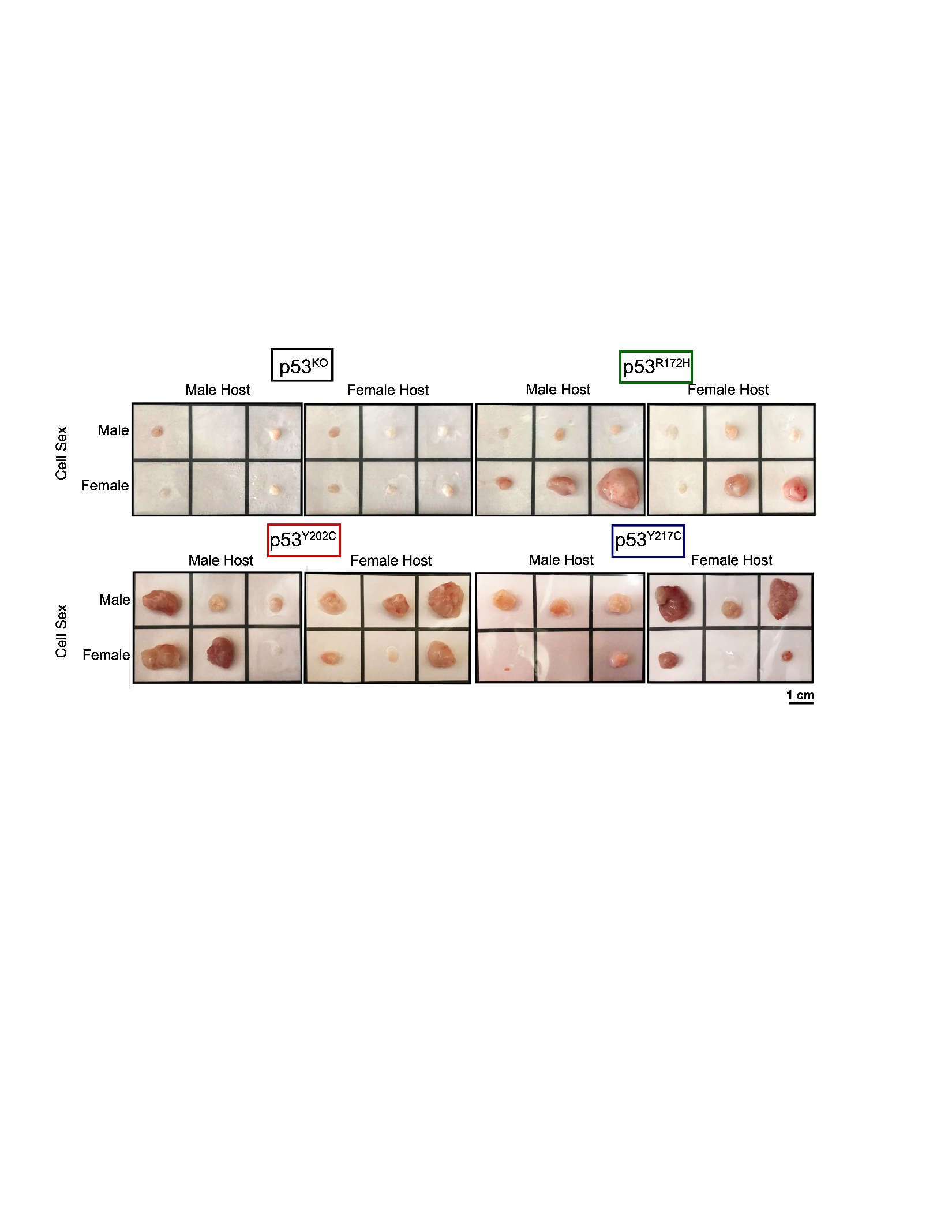
**

**Supplemental Figure 2.** Images of harvested flank tumors or recovered Matrigel pellet from mice injected with male and female p53 KO (black), p53^R172H^ (green), p53^Y202C^ (red), and p53^Y217C^ (blue). Where blank squares are present, no tumor or Matrigel pellet was recovered.

**
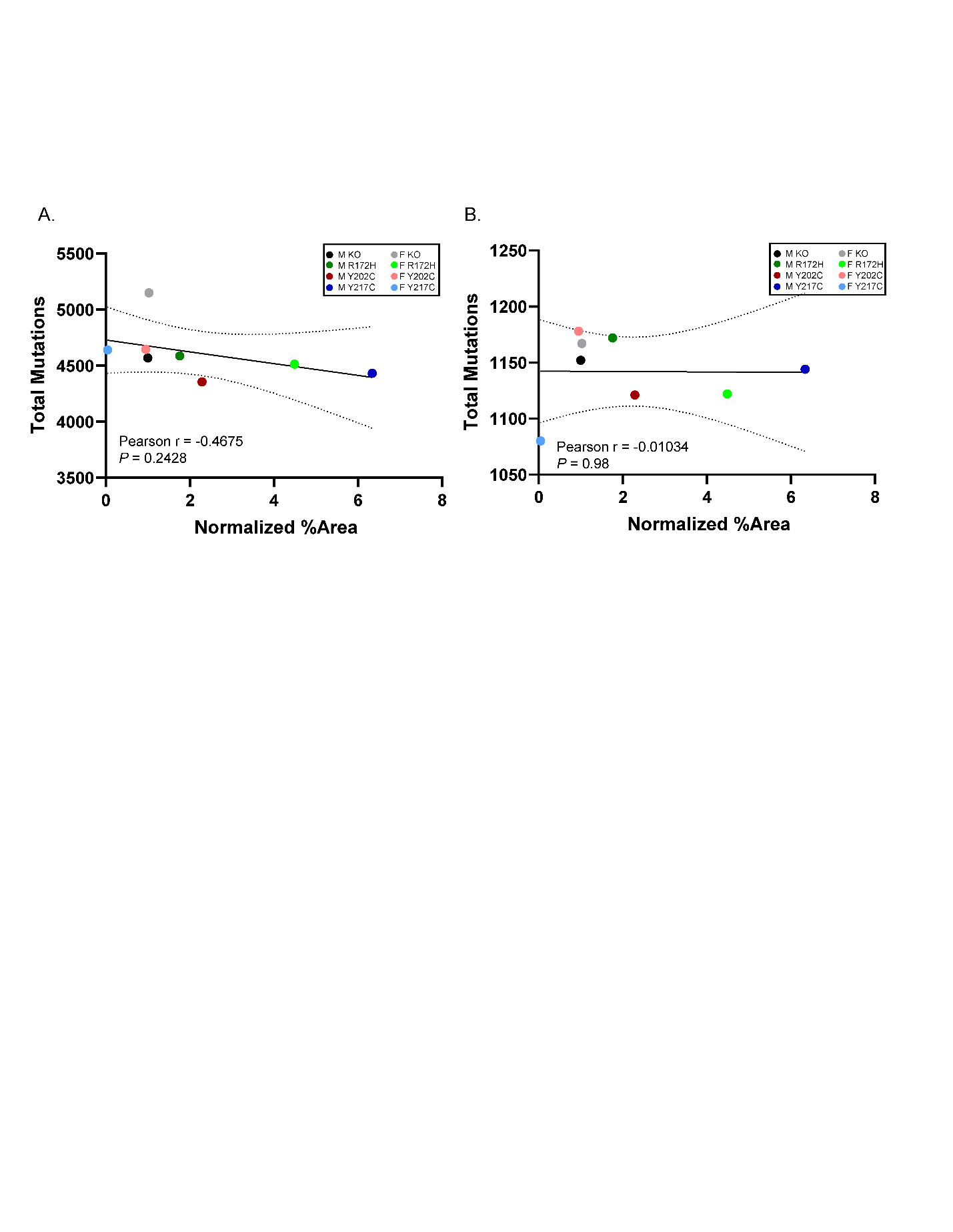
**

**Supplemental Figure 3.** Cell growth phenotype does not correlate with mutation burden. Pearson correlation of the relationship between growth as measure by normalized percent area from the foci assay and the total number of mutations (**A**) or the number of missense or nonsense mutations (**B**) identified in whole exome sequencing.

**Supplemental Table 2.** All identified mutant genes in male or female p53 KO, p53^R172H^, p53^Y202C^, and p53^Y217C^ astrocytes. A (+) indicates a homologous mutation in the given gene in the corresponding mutation p53 cell line.

**

**

**




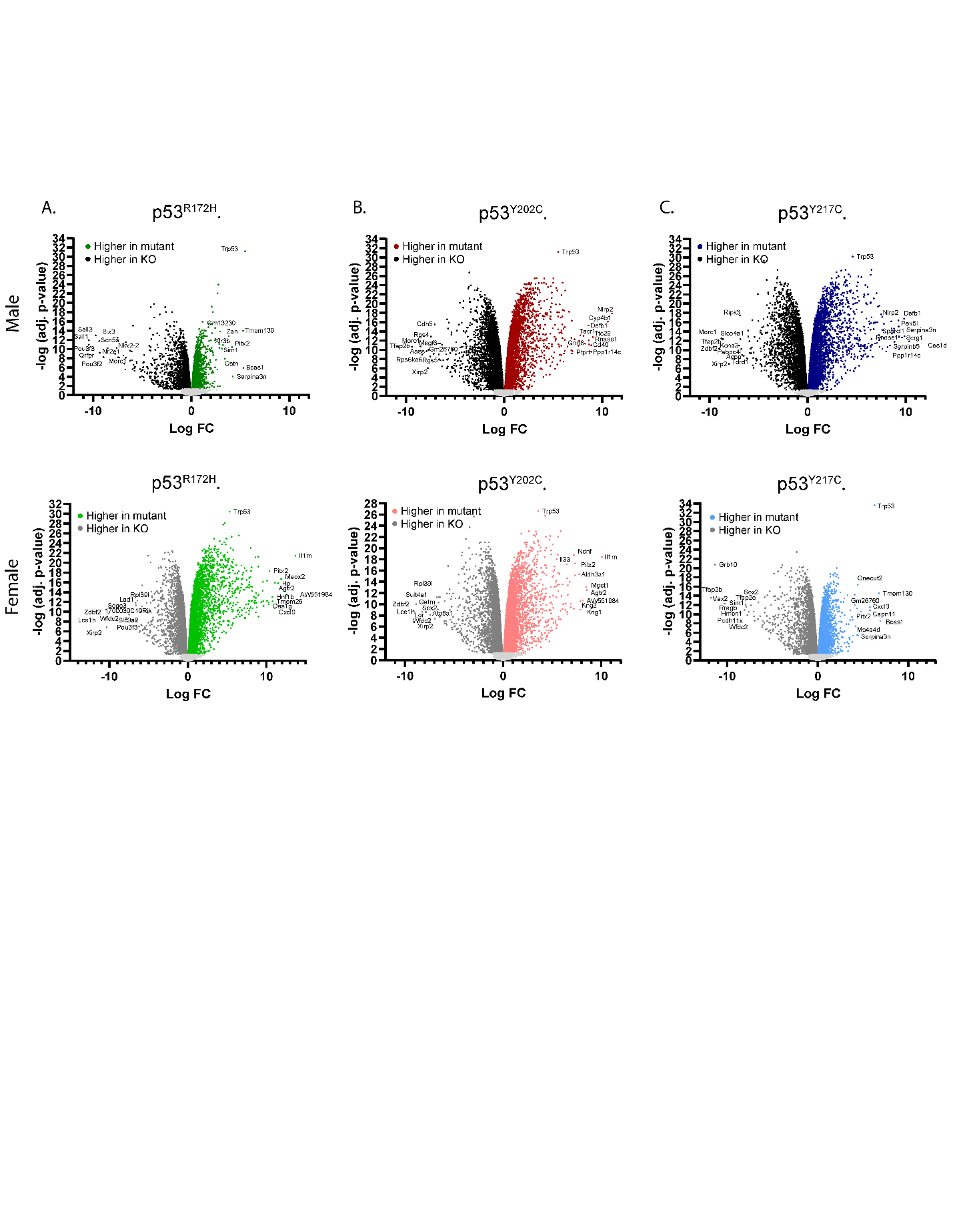
**

**Supplemental Figure 4.** Volcano plots displaying all significant differential gene expression (adjusted p-value < 0.05) between mutant p53 and p53 KO astrocytes within each sex for **A.** p53^R172H^ (green), **B.** p53^Y202C^ (red), **C.** p53^Y217C^ (blue). The top ten upregulated and downregulated genes by logFC and *Trp53* are labeled in each plot. Genes below the significance threshold are colored light grey.


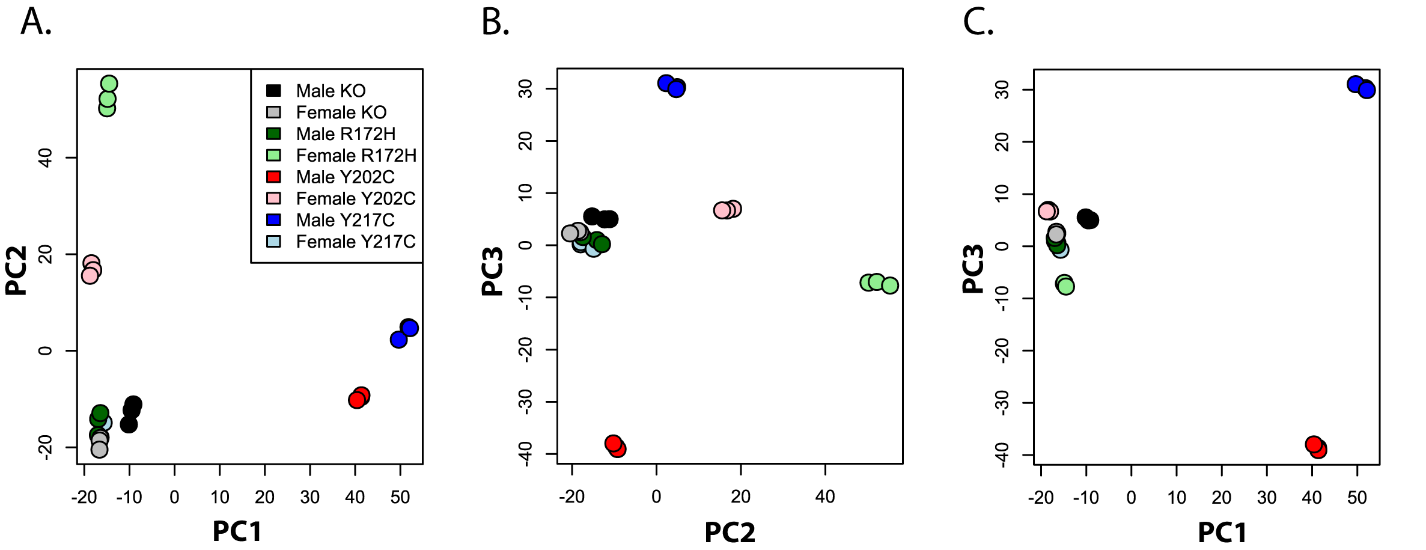


**Supplemental Figure 5**. 2-dimensional representation of sparse PCA analysis. **A)** PC1 vs PC2 **B)** PC2 vs PC3 **C)** PC1 vs PC3.

**Supplementary Table 3.** Comparison of the total number (n) of genes identified with a significant interaction between sex and mutation and all significant differentially expressed genes with a log_2_(FC) > 0.5 (FDR < 0.05).

|  | **Interaction** | **Male-only**  **DE genes** | **Female-only**  **DE genes** | **Overlap (Male)** | **Overlap (Female)** |
| --- | --- | --- | --- | --- | --- |
| **R172H** | **9287** | **581** | **4305** | **432 (74%)** | **3907 (91%)** |
| **Y202C** | **10441** | **3416** | **2517** | **3093 (91%)** | **2211 (88%)** |
| **Y217C** | **9387** | **3583** | **1376** | **3168 (88%)** | **1154 (84%)** |

**Supplementary Table 4. KEGG pathway enrichment analysis overlapping genes from the interaction modeling and differential expression analysis: p53^R172H^.**

**Male: No significant enrichment**

**Female:**

| **Enrichment FDR** | **Genes in list** | **Total genes** | **Functional Category** |
| --- | --- | --- | --- |
| 0.00011 | 45 | 123 | Cell cycle |
| 0.00032 | 31 | 78 | Ribosome biogenesis in eukaryotes |
| 0.00032 | 45 | 131 | Lysosome |
| 0.00032 | 26 | 61 | Fatty acid metabolism |
| 0.0038 | 327 | 1546 | Metabolic pathways |
| 0.0038 | 16 | 35 | DNA replication |
| 0.0038 | 42 | 136 | Apoptosis |
| 0.0038 | 57 | 201 | Proteoglycans in cancer |
| 0.0038 | 48 | 162 | MicroRNAs in cancer |
| 0.0047 | 48 | 165 | RNA transport |
| 0.0047 | 56 | 200 | Focal adhesion |
| 0.0065 | 51 | 181 | Transcriptional misregulation in cancer |
| 0.0074 | 49 | 174 | Cellular senescence |
| 0.0074 | 42 | 143 | Fluid shear stress and atherosclerosis |
| 0.0086 | 48 | 171 | Hepatocellular carcinoma |
| 0.01 | 57 | 214 | Rap1 signaling pathway |
| 0.01 | 124 | 537 | Pathways in cancer |
| 0.012 | 24 | 71 | P53 signaling pathway |
| 0.012 | 24 | 71 | Adherens junction |
| 0.012 | 29 | 92 | Small cell lung cancer |
| 0.014 | 16 | 41 | Homologous recombination |
| 0.021 | 72 | 294 | MAPK signaling pathway |
| 0.023 | 19 | 55 | Valine, leucine and isoleucine degradation |
| 0.023 | 13 | 32 | Beta-Alanine metabolism |
| 0.023 | 30 | 101 | AGE-RAGE signaling pathway in diabetic complications |
| 0.031 | 13 | 33 | Base excision repair |
| 0.033 | 55 | 219 | Salmonella infection |
| 0.034 | 32 | 113 | TNF signaling pathway |
| 0.037 | 58 | 235 | Human T-cell leukemia virus 1 infection |
| 0.037 | 17 | 50 | Fatty acid degradation |

**Supplementary Table 5.** KEGG pathway enrichment analysis overlapping genes from the interaction modeling and differential expression analysis: p53^Y202C^.

**Male:**

| **Enrichment FDR** | **Genes in list** | **Total genes** | **Functional Category** |
| --- | --- | --- | --- |
| 0.00015 | 47 | 163 | Protein processing in endoplasmic reticulum |
| 0.002 | 37 | 131 | Lysosome |
| 0.0057 | 18 | 49 | Amino sugar and nucleotide sugar metabolism |
| 0.019 | 63 | 294 | MAPK signaling pathway |
| 0.034 | 258 | 1546 | Metabolic pathways |
| 0.034 | 28 | 107 | Toxoplasmosis |
| 0.037 | 47 | 214 | Rap1 signaling pathway |
| 0.04 | 16 | 51 | Fanconi anemia pathway |
| 0.04 | 36 | 156 | MTOR signaling pathway |
| 0.04 | 28 | 113 | TNF signaling pathway |
| 0.04 | 47 | 219 | Salmonella infection |
| 0.043 | 32 | 136 | Apoptosis |

**Female:**

| **Enrichment FDR** | **Genes in list** | **Total genes** | **Functional Category** |
| --- | --- | --- | --- |
| 0.00074 | 206 | 1546 | Metabolic pathways |
| 0.00074 | 45 | 227 | Thermogenesis |
| 0.046 | 26 | 131 | Oxidative phosphorylation |

**Supplementary Table 6.** KEGG pathway enrichment analysis overlapping genes from the interaction modeling and differential expression analysis: p53^Y217C^.

**Male:**

| **Enrichment FDR** | **Genes in list** | **Total genes** | **Functional Category** |
| --- | --- | --- | --- |
| 0.0000017 | 45 | 131 | Lysosome |
| 0.00006 | 288 | 1546 | Metabolic pathways |
| 0.00019 | 116 | 537 | Pathways in cancer |
| 0.00034 | 47 | 171 | Hepatocellular carcinoma |
| 0.0004 | 55 | 214 | Rap1 signaling pathway |
| 0.0016 | 30 | 99 | Prostate cancer |
| 0.0019 | 50 | 201 | Proteoglycans in cancer |
| 0.003 | 39 | 148 | Gastric cancer |
| 0.003 | 45 | 180 | Axon guidance |
| 0.0042 | 41 | 162 | MicroRNAs in cancer |
| 0.0053 | 17 | 48 | ABC transporters |
| 0.0053 | 26 | 89 | Fc gamma R-mediated phagocytosis |
| 0.0065 | 17 | 49 | Amino sugar and nucleotide sugar metabolism |
| 0.011 | 19 | 61 | Fatty acid metabolism |
| 0.011 | 40 | 167 | Phagosome |
| 0.011 | 72 | 348 | Human papillomavirus infection |
| 0.011 | 21 | 70 | Acute myeloid leukemia |
| 0.011 | 20 | 66 | Non-small cell lung cancer |
| 0.013 | 51 | 231 | Ras signaling pathway |
| 0.015 | 12 | 32 | Galactose metabolism |
| 0.015 | 12 | 32 | Beta-Alanine metabolism |
| 0.016 | 12 | 33 | Propanoate metabolism |
| 0.016 | 32 | 131 | FoxO signaling pathway |
| 0.016 | 23 | 84 | Peroxisome |
| 0.016 | 30 | 120 | Thyroid hormone signaling pathway |
| 0.016 | 28 | 109 | Insulin resistance |
| 0.016 | 35 | 147 | Breast cancer |
| 0.018 | 26 | 101 | AGE-RAGE signaling pathway in diabetic complications |
| 0.021 | 15 | 48 | Sphingolipid metabolism |
| 0.021 | 24 | 93 | Endocrine resistance |
| 0.021 | 36 | 156 | MTOR signaling pathway |
| 0.021 | 70 | 353 | PI3K-Akt signaling pathway |
| 0.021 | 47 | 219 | Salmonella infection |
| 0.021 | 17 | 58 | Endometrial cancer |
| 0.028 | 36 | 160 | Cushing syndrome |
| 0.029 | 16 | 55 | Valine, leucine and isoleucine degradation |
| 0.029 | 43 | 200 | Focal adhesion |
| 0.032 | 36 | 162 | Hepatitis B |
| 0.035 | 23 | 92 | Small cell lung cancer |
| 0.038 | 20 | 77 | Synaptic vesicle cycle |
| 0.04 | 36 | 165 | Tight junction |
| 0.04 | 27 | 115 | Leukocyte transendothelial migration |
| 0.041 | 32 | 143 | Fluid shear stress and atherosclerosis |
| 0.047 | 20 | 79 | EGFR tyrosine kinase inhibitor resistance |

**Female:**

| **Enrichment FDR** | **Genes in list** | **Total genes** | **Functional Category** |
| --- | --- | --- | --- |
| 0.018 | 23 | 180 | Axon guidance |

**Supplementary Table 7**. Comparison of the total number of genes (n) identified with a significant interaction between mutation and gene expression and all shared significant differentially expressed genes with a log_2_(FC) > 0.5 (FDR < 0.05).

|  | **Marginal effect in**  **interaction test** | **Shared DE genes**  **in both sexes** | **Overlap** |
| --- | --- | --- | --- |
| **R172H** | **5639** | **1314** | **1306 (99%)** |
| **Y202C** | **5568** | **3024** | **3001 (99%)** |
| **Y217C** | **3670** | **2295** | **2262 (99%)** |
